# Elastic MCF Rubber with Photovoltaics and Sensing on Hybrid Skin (H-Skin) for Artificial Skin by Utilizing Natural Rubber: Third Report on Electric Charge and Storage under Tension and Compression †

**DOI:** 10.3390/s18061853

**Published:** 2018-06-06

**Authors:** Kunio Shimada

**Affiliations:** Faculty of Symbiotic Systems Sciences, Fukushima University, 1 Kanayagawa, Fukushima 960-1296, Japan; shimadakun@sss.fukushima-u.ac.jp; Tel.: +81-24-548-5214

**Keywords:** hybrid skin (H-Skin), solar cell, battery, charge, discharge, sensing, piezoelectricity, photovoltaics, natural rubber, electrolytic polymerization, magnetic cluster, magnetic field, magnetic compound fluid (MCF), artificial skin, robot, humanoid

## Abstract

In the series of studies on new types of elastic and compressible artificial skins with hybrid sensing functions, photovoltaics, and battery, we have proposed a hybrid skin (H-Skin) by utilizing an electrolytically polymerized magnetic compound fluid (MCF) made of natural rubber latex (NR-latex). By using the experimental results in the first and second reports, we have clarified the feasibility of electric charge at irradiation, and that without illumination under compression and elongation. The former was explained in a wet-type MCF rubber solar cell by developing a tunneling theory together with an equivalent electric circuit model. The latter corresponds to the battery rather than to the solar cell. As for the MCF rubber battery, depending on the selected agent type, we can make the MCF rubber have higher electricity and lighter weight. Therefore, the MCF rubber has an electric charge and storage whether at irradiation or not.

## 1. Introduction

Artificial skin will be required more in the future as a substitute for human skin and as a part of the humanoid exterior of robots. The requisites for artificial skin are the material properties of flexibility, elasticity, and extensibility, and a high sensing ability for force and temperature. The former requires high tension and compression for sturdiness. The latter is related to haptic sensing, as human skin has five types of touch sensation: tactile, pressure sensation, algometry, warm, and cold [1]. Current ordinary sensors have a unique sensitivity to forces that are applied normal to a touched material; however, they do not have sensitivity to a shear force. When we need to measure the shear force, strain gauges or piezoelectric elements must be added [2,3,4,5], making the sensor’s structure complicated and vulnerable to extrinsic mechanical forces. Therefore, it is significant to make the sensor more sensitive with a simple morphology. The sensing of shear force is effective in the case of rubbing some object, such as for example when measuring surface roughness [6], the softness of a soft material such as a diaper [7], or the case of a robot stroking a person’s head.

On the other hand, current ordinary robots require a lightweight battery. If the battery is not incorporated into the robot, electric wires connected to the outlet of an electric source are required, and can act as an obstacle. There have been proposed lightweight batteries, solar cells, etc. However, recently, batteries, including polymer batteries, have been developed. If the skin installed in a robot has a battery, the battery must be elastic and compressible in addition to being more lightweight. A battery made of rubber has been investigated [8,9]. However, the effects of compression and elongation on the conductivity properties have not been clarified. To answer these problems, a solar cell is proposed to be installed in a robot, usually in the form of a solid-state flat plate. Although flexible solar power generators have been investigated recently, including a conducting polymer [10,11,12,13,14,15,16], they have remained within the realm of solid-state devices. Based on the above, the utilization of a rubber in batteries and solar cells is a significant step toward solving the problems related to the material dynamic properties of elasticity, compressibility, and weight.

In order to make the rubber utilized in batteries and solar cells more sensitive, as described above, we have proposed a lightweight hybrid skin (H-Skin) integrated with multiple functionalities of flexibility, elasticity, and compressibility, photovoltaics, and high sensing modalities for force and temperature for artificial human skin, humanoid skin, or the outer layer of robot skin in the first report [17]. On this subject, we have investigated temperature susceptibility and challenged the sensing of various bodily surfaces related to shearing motion by dry-type magnetic compound fluid (MCF) rubber, as shown in Appendix A, before the series of these present three reports. Dry-type MCF rubber means that the rubber was solidified in air without electrolytic polymerization. In contrast, electronic skin (E-Skin) has been proposed by integrating multiple functionalities of sensing modalities to force, temperature, and so on for sensitive skin, smart skin, or intelligent skin [18]. The difference between H-skin and E-Skin is that the former has photovoltaic functionality, whereas the latter does not, as presented previously by the first report [17]. Since H-Skin also has piezoelectricity, it can use solar power for self-sensing by utilizing the generated piezo effect. Piezoelectricity corresponds to the piezoelectric effect, which is the built-in voltage generated by the approaching of positive and negative ions, and is different from piezoresistivity, which means changes in resistance (piezoresistive resistance) when a voltage is applied under compression or elongation.

We have investigated H-Skin for its properties of photoelectricity (photovoltage and photocurrent) as a solar cell and built-in electricity (built-in voltage and built-in current) from its piezoelectricity by utilizing the MCF rubber used in the previous two reports [17,19]. MCF rubber is the elastic and compressible rubber included in the MCF, which is an intelligent fluid that is responsive to a magnetic field involving 10-nm Fe_3_O_4_ particles coated by oleic acid because of the compounding magnetic fluid (MF) and other metal particles, such as Fe, Ni, or Cu, on the order of 1 μm. MF is a significant factor in producing MCF rubber; the role of magnetic fluid (MF) corresponds to the one oleic acid coated around the Fe_3_O_4_ particle. The dielectric polarization of the oleic acid is significant. The dielectricity induces the built-in voltage and current, which is generated as another mechanism by the gap between A^−^ and D^+^. The built-in electricity and photoexcitation are based on *p*-type and *n*-type semiconductors viewing in the isoprene molecules of NR-latex, oleic acid, water, and Ni particles, photosensitized dye molecules, and so on. By ionized *p*-type and *n*-type semiconductors, the former becomes an acceptor A, which is charged negatively to become A^-^, and the latter becomes a donor D, which is charged positively to become D^+^. In addition, the production of MCF rubber by the electrolytic polymerization is significant for solidification among the isoprene of the NR-latex particle and the oleic acid. This mechanism has been clarified by the previous report [20]. The MCF rubber consisting of natural rubber (NR-latex) is solidified under the application of a magnetic field, and magnetic clusters combined by the aggregation of Fe_3_O_4_ and metal particles are formed along the direction of the magnetic field lines. The first report showed that MCF rubber can become a solar cell without dye or electrolytes. In general, ordinary organic thin solar cells have sensitized dye and electrolytes for photoexcitation by the oxidation–reduction reaction. In addition, the electrolytically polymerized MCF rubber involving the dye and electrolyte was also able to become a solar cell. These cases were the dry-type MCF rubber solar cell. The type includes another condition in which dye and electrolyte are poured on an MCF rubber dried in a drying machine, which was also able to become a solar cell in the second report. The first report showed the physical–chemical fabrication and principle of the dry-type solar cell, and clarified the effect of compression on its photoelectricity. In contrast, the second report dealt with the wet-type solar cell, which denotes pouring dye and electrolyte. The same physical–chemical fabrication and principle of the solar cell as in the dry-type MCF rubber solar cell were established in the wet-type cell. This clarified the effects of several experimental condition factors, namely, of the simultaneous existence of both compression and elongation on its photoelectricity and piezoelectricity, and the electrode location.

However, the electric charging of the MCF rubber has not been elucidated. Since electricity can be charged between A^−^ and D^+^, MCF rubber can be proposed to create a feasible battery.

Therefore, in the present study, we clarify the electric charging of wet-type MCF rubber solar cells. Furthermore, the effects of compression and elongation on the electric charging are clarified. In addition, the MCF rubber without irradiation is found to have the function of electric charging, which corresponds to a battery without sensitized dye. Its effects of compression and elongation on the electric charging of the MCF rubber battery are also clarified.

## 2. Photovoltaics MCF Rubber Solar Cell

**Figure 1:**
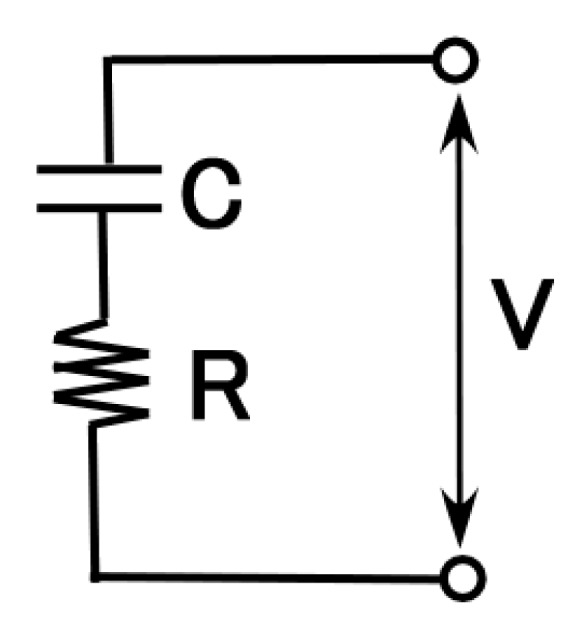
Equivalent electric circuit with capacitance *C* and resistance *R* of a magnetic compound fluid (MCF) rubber solar cell.

An ordinarily solar cell is evaluated with an equivalent electric circuit comprising a capacitor and resistor, as shown by Figure 1. First, we investigate the resistance *R*, and the total resistance of the solar cell in which there exists some internal resistances.

**Figure 2:**
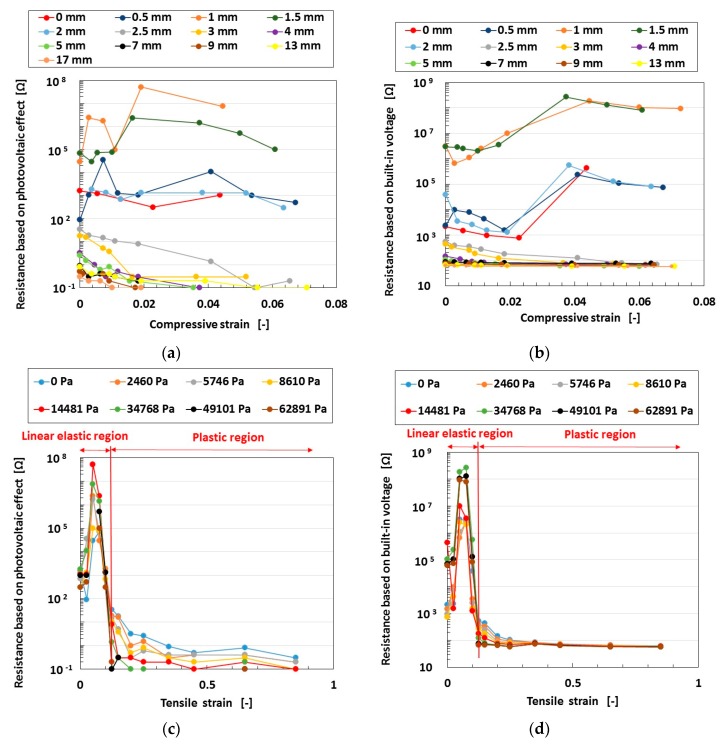
Changes in the effect of compression and elongation on resistance based on the photovoltaic effect and built-in electricity under ultraviolet light. (**a**,**b**) under compression; (**c**,**d**) under elongation; (**a**,**c**) based on photovoltaic effect; (**b**,**d**) based on built-in electricity for a wet-type MCF rubber solar cell electrolytically polymerized under a magnetic field with 0.22 g of Ruthenium complex dye and 7.4 g of KI+I_2_ electrolyte. The MCF rubber had 3 g of TiO_2_, 6 g of Ni, 4.5 g of MF, and 9 g of natural rubber (NR)-latex. The irradiation light was ultraviolet (40 lx).

The experimental data of *R* in a wet-type MCF rubber solar cell is shown in Figure 2 under elongation and compression. Figure 2a,b shows the amplitude of tension and Figure 2c,d shows that of pressure. The experimental apparatus and procedure were the same as those used in the second report [19]. In general, the voltage and current of the solar cell have both photo and built-in electricity. The former and the latter causes were explained previously in the second report. Therefore, resistance can be estimated to be divided between the two cases. At a lesser elongation, by compression, the photovoltaic resistance is almost constant; however, the built-in voltaic resistance increases. At a greater elongation, by compression, both resistances decrease or remain constant. On the other hand, by elongation, both resistances have a peak in the linear elastic region of the MCF rubber, and then decrease in the plastic region. In contrast, tunneling theory is applicable to the MCF rubber solar cell. The transfer of electric current over the potential barrier of the rubber under compression as a one-dimensional Schrödinger equation is easier because of the decreasing size of the gap of the insulator, as shown previously in the first report [17]. From the equations presented in the report, the resistance *R* of the MCF rubber solar cell is given by Equation (1) with a transmitted probability of *T*, where *eE_o_* is the applied voltage, *e* is the elementary charge, *γ* is the wave number presented by Equation (2), *ħ* is *h/2*π, *h* is Planck’s constant, *m* is the mass of the electron, *V_o_* is the potential energy at region *γ* for each *i*-ordered pair of regions fabricated with non-conductive rubber sandwiched by conductive materials, and *ε* is the energy of the electron. At each *i*-ordered pair of regions, the transmitted current is given as the denominator of the middle expression of Equation (1) and approximately evaluated as *T_out_*, which is *T* at the outlet of *n* pairs of regions through which the electrons transfer.

(1) $$R=\frac{eE_{o}}{\sum_{i=1}^{n}T_{i}\left(\frac{\gamma_{i}}{\gamma_{i+1}}-\frac{\gamma_{i+1}}{\gamma_{i}}\right)}≅\frac{eE_{o}}{T_{out}}$$

(2) $$\gamma =\sqrt{\frac{2m}{\hbar^{2}}(V_{o}-\varepsilon )}$$

**Figure 3:**
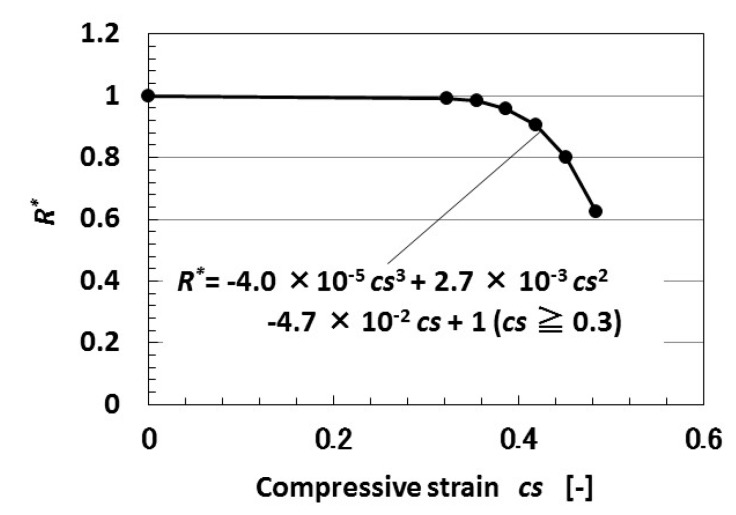
Theoretical results of dimensionless resistance to compression by tunneling theory.

As a result, dimensionless resistance R* (=*R/R_∞_*, *R_∞_* the representative resistance) to compressive strain *cs* is given by Figure 3. At a high tensile strain, the resistances based on both the photovoltaic effect and built-in voltage decrease with increasing compressive strain. The tendencies in Figure 2a,b can be explained by the theoretical results of Figure 3. However, the properties of these resistances in Figure 2c,d at low tensile strain cannot be explained by the present tunneling theory, and are due to the complicated fabrication of particles involved in the MCF rubber solar cell.

**Figure 4:**
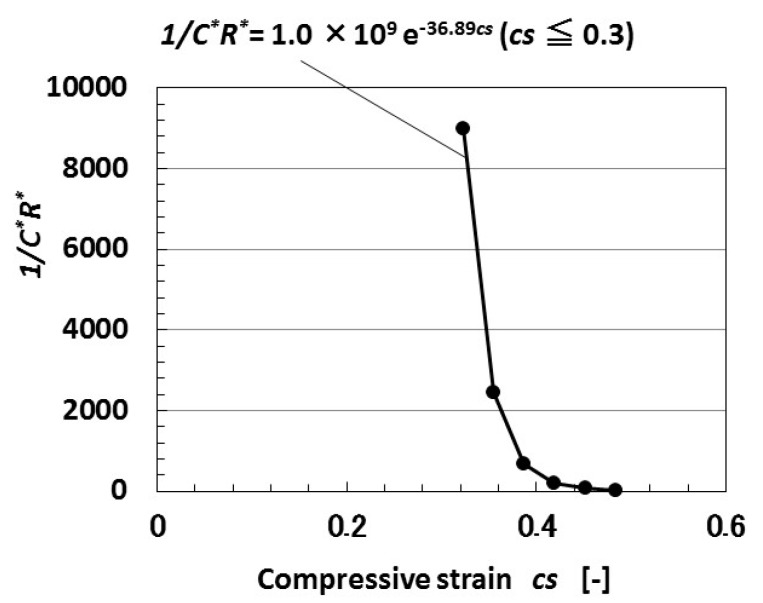
Theoretical results of 1/C*R* to compression by tunneling theory.

Second, we investigate the parameters of *C* and *R*. As metal particles facing each other can be assumed to have the same charge as the parts of an *n*–*p*-type semiconductor, the dimensionless capacitance C_n_* located at *n*’s turn is presented as Equation (3), where *ε’* is the dielectric constant of the MCF rubber solar cell. The dimensionless capacitance C* of the MCF rubber solar cell is calculated as that at the outlet of *n* pairs of regions through which the electron transfers. As a result, 1/C*R* is given by Figure 4 as an exponential function of *cs*.

(3) $${C_{n}}^{*}=\frac{{\varepsilon^{\prime }}^{n}}{2\sum_{J=1}^{n}\frac{1+T_{j}^{3/4}}{{\left(\prod_{i=1}^{j}T_{i}\right)}^{3/4}}}$$

On the other hand, electric current *I* in the circuit of Figure 1 is presented as Equation (4) with dimensionless factors of C* and R*, where t* is the dimensionless time. The formula of Equation (4) presents that *I* is an exponential function of 1/C*R*.

(4) $$I=\frac{V}{R}\exp\left(\frac{t^{*}}{C^{*}R^{*}}\right)$$

**Figure 5:**
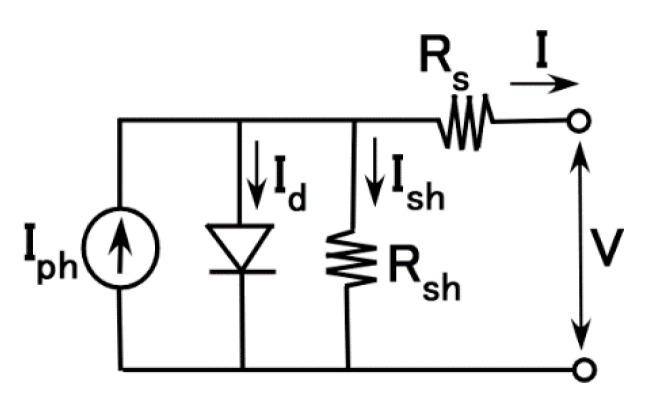
Equivalent electric circuit of MCF rubber solar cell.

By substituting the equation for 1/C*R* shown in Figure 4 to Equation (4), we find that *I* contains an exponential function of *cs*, and that *I* decreases with increasing *cs*. The theoretical results qualitatively coincide with the experimental results obtained in the second report [19], in which the built-in current and photocurrent density decrease with increasing compressive strain. This tendency implies that the isolated layers of rubber or oleic acid among the particles and molecules in the *n*–*p*-type semiconductor dispersed in the MCF rubber solar cell are changed by compression. Therefore, we suggest that there exist thin layers among them. Based on this suggestion, we propose that *I* is presented by Equation (5) in the case of another equivalent electric circuit; it is usually introduced in ordinary solar cells as shown by Figure 5, where *I_S_* is the reverse-bias saturation current, *N_d_* is the diode ideality factor (=*V_f_*/(*V_f_* − *V_i_*)), *V_f_* is the forward bias voltage, *V_i_* is the voltage drop at the thin isolated layer, *k* is the Boltzmann constant, *T_a_* is the absolute temperature, *I_ph_* is the photocurrent, *I_d_* is the diode current, *I_sh_* is the shunt current, *R_sh_* is the shunt resistance, and *R_s_* is the series resistance. *N* denotes recombination in the *n*–*p*-type semiconductor; therefore, we can guess that in some of the *n*-type and *p*-type semiconductors of isoprene, oleic acid, and water molecules, the Ni particles recombine.

(5) $$I=I_{S}\exp\left(\frac{eV_{f}}{N_{d}kT_{a}}\right)$$

As a result, we can explain the photovoltaic property of the MCF rubber solar cell under elongation and compression by utilizing the equivalent electric circuit models shown in Figure 1 and Figure 5 as follows. 

The isolated layers of rubber or oleic acid among the particles and molecules are not always thin; therefore, *I* is generally presented by Equation (6) from the Shockley diode equation and Ohm’s law. As seen from the middle expression of Equation (6), the experimental data of the electric current *I* includes the photocurrent *I_ph_* and built-in current, which depends on *I_d_*, and can be negative by the quantitative balance of *I_ph_*, *I_d_*, and *I_sh_* under irradiation, as was presented in the previous two reports [17,19]. *I_d_* implies the dark current and is relevant to *R_sh_*. The dark current is generated by the influx of holes and electrons, which results from the existence of the depletion layer between the *n*-type and *p*-type semiconductors. *I_d_* is a function of the diffusion potential *V_D_*, which is presented by the difference in electron affinity between the *n*-type and *p*-type semiconductors. The difference in electron affinity denotes the Schottky barrier between heterogeneous materials. Therefore, by considering, in addition to the experimental results, that *I* decreases with increasing *cs*, the potential and electron affinity among isoprene, oleic acid, water molecules, and Ni particles are different enough to become a part of the semiconductor. In addition, the difference in potential and electron affinity, or *V_D_*, changes by compression or elongation. Therefore, the photo and built-in currents change by compression or elongation, as shown in the previous reports [17,19].

(6) $$I={I_{ph}-I_{d}-I_{Sh}=I}_{ph}-I_{S}\left[\exp\left\{\frac{e\left(V+R_{S}I\right)}{NkT_{a}}\right\}-1\right]-\frac{V+R_{S}I}{R_{Sh}}$$

**Figure 6:**
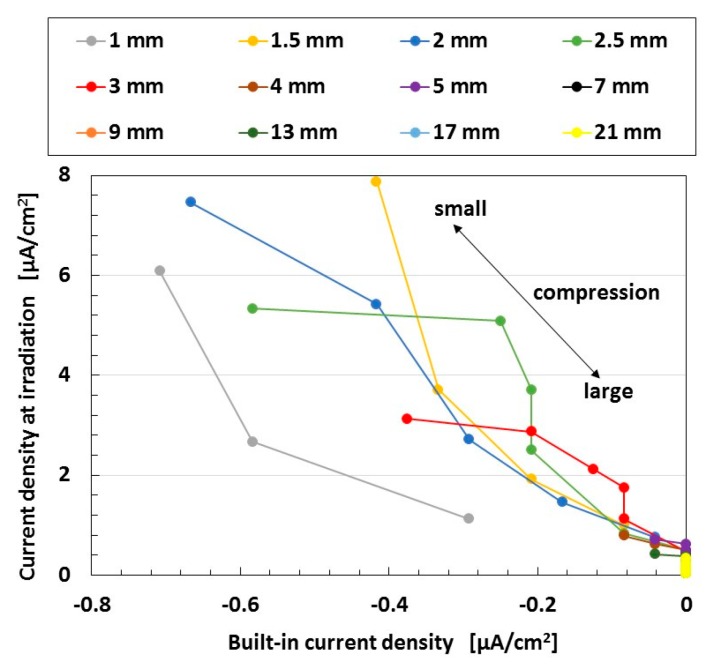
Relation between current density at irradiation and built-in current density under compression and elongation under ultraviolet light for a wet-type MCF rubber solar cell electrolytically polymerized under a magnetic field with 0.22 g of Ruthenium complexes dye and 7.4 g of KI+I_2_ electrolyte. The MCF rubber had 3 g of TiO_2_, 6 g of Ni, 4.5 g of MF, and 9 g of NR-latex. The irradiation light was ultraviolet (40 lx). The experimental apparatus and procedure were the same as those used in the second report [19].

On the other hand, NR-latex particles and oleic acid coated around Fe_3_O_4_ in MCF rubber can also be considered to have a spontaneous polarization of the dielectric. In that case, as the well-known formula at the field of ferroelectric material, the photocurrent *I_ph_* is presented to be approximated to *J* by Equation (7) as a function of glass factor *k*_1_, which is dependent on the amplitude of spontaneous polarization, where *α* is the optical absorption coefficient, *f* is the frequency of light, and *I_r_* is the light intensity [21]. This formula has been used at the case of explanation of anomalous photovoltaic effect, which is also called the bulk photovoltaic effect. As *k*_1_ is considered to be involved in the built-in current, the relation between the photo and built-in currents is linear, which coincides with the relation between the current at irradiation and the built-in current from the experimental data of the second report [19], as shown in Figure 6. In the figure, each line at each amplitude of tension is the change due to compression. As the compression increases, these currents decrease because of increased contact between the NR-latex particles and oleic acid as the distance between them decreases.

(7) $$J=\frac{k_{1}e\alpha I_{r}}{2\pi f\hbar }$$

**Figure 7:**
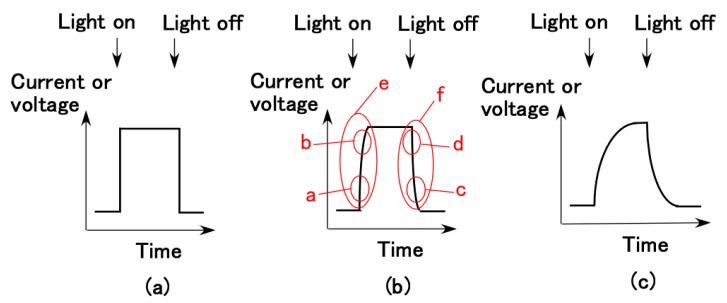
Schematic response curve of voltage and electric current by light-on and off: (**a**) no time lag; (**b**) dominant with *t*_1_ or *t*_3_; (**c**) dominant with *t*_2_.

On the other hand, in the case of the equivalent electric circuit from Figure 1, *C R* presents the time constant, which results in the degree of the rising and falling edges of the current or voltage curves as shown by Figure 7, and which indicates the response time for irradiation. The response time is minimal enough to behave as in Figure 7a. However, wet-type solar cells, such as dye-sensitized solar cells, have a delay to irradiation because of the oxidation–reduction reaction. The response time *t* is generally divided into three types, as shown in Equation (8): *t*_1_ based on *C* and *R* corresponding to Figure 7b; *t*_2_ based on the speed of carrier diffusion at deeper regions than the depletion layer corresponding to Figure 7c; and *t*_3_ based on the speed of carriers in the inner depletion layer corresponding to Figure 7b.

(8) $$t=\sqrt{t_{1}^{2}+t_{2}^{2}+t_{3}^{2}}$$

**Figure 8:**
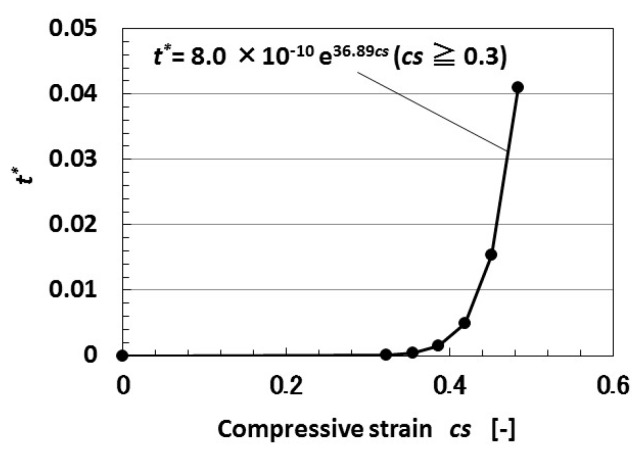
Theoretical results of dimensionless time constant to compression by tunneling theory.

**Figure 9:**
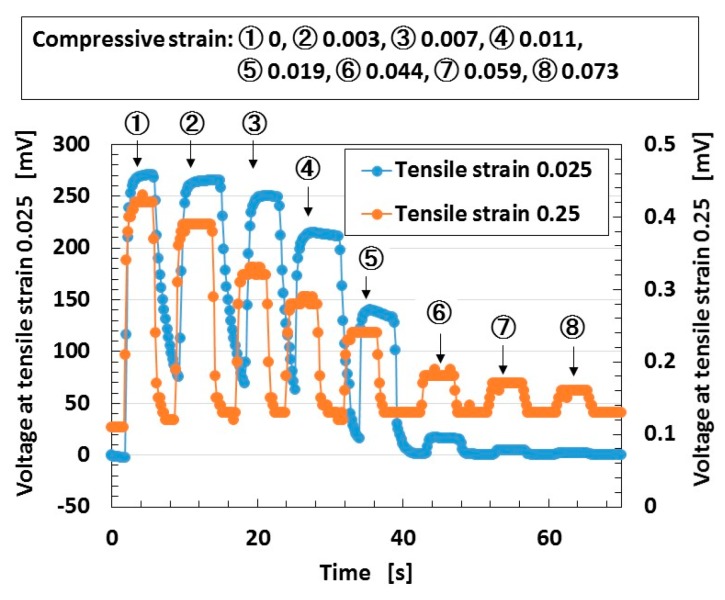
Change in voltage by repetition of compression under elongation under ultraviolet light for a wet-type MCF rubber solar cell electrolytically polymerized under a magnetic field with 0.22 g of Ruthenium complexes dye and 7.4 g of KI+I_2_ electrolyte. The MCF rubber had 3 g of TiO_2_, 6 g of Ni, 4.5 g of MF, and 9 g of NR-latex. The irradiation light was ultraviolet (40 lx). The experimental apparatus and procedure were the same as those used in the second report [19]. ① to ⑧ indicate each degree of compressive strain.

Regarding *t*, the theoretical result of the dimensionless time constant t* (=C*R*) by the tunneling effect obtained from Equations (1) and (3) is shown by Figure 8. t* increases with increasing *cs*. In contrast, the experimental result of the voltage in wet-type MCF rubber solar cells under both elongation and compression is shown by Figure 9.

Under tensile strains of 0.025 and 0.25, the MCF rubber solar cell was gradually compressed with increasing compression strain from ① to ⑧. The tendency as shown by Figure 7c can be seen under smaller compressive strain, and the tendency changes to Figure 7a through Figure 7b with increasing compressive strain. This denotes that by the compression of the characteristics of reacting carriers of *n*-type and *p*-type semiconductors of isoprene, oleic acid, and water molecules, Ni particles change from state *t*_2_ to states *t*_1_ and *t*_3_, and states *t_1_* and *t_3_* depreciate. The theoretical result of Figure 8 correspond to the change in *t_1_* by compression. On the other hand, when the tensile strain increases, the tendency changes from Figure 7c to Figure 7b. This indicates that by elongation, the characteristics of the reacting carriers change from state *t*_2_ to state *t*_1_ or *t*_3_.

**Figure 10:**
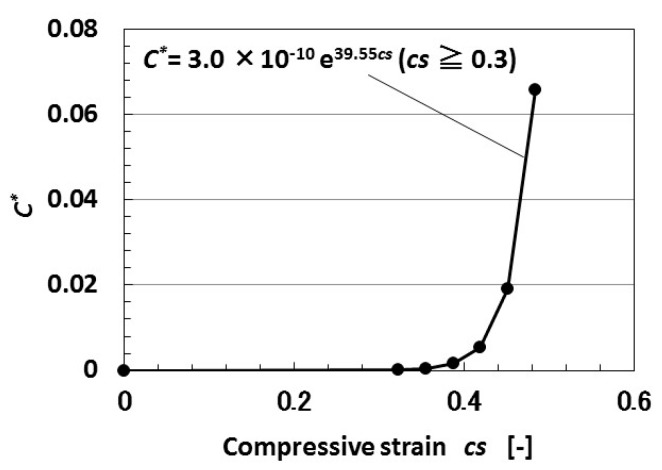
Theoretical results of dimensionless capacitance to compression by tunneling theory.

Finally, we investigate the charge of the MCF rubber solar cell by irradiation. The theoretical result of C* by the tunneling effect obtained from Equations (1) and (3) is shown in Figure 10. From the results of Figure 2, Figure 7 and Figure 8 under irradiation, we can also obtain the same result that *C* increases with increasing compression, as shown in Figure 10.

On the other hand, as shown in Figure 7b, *t* changes as the light is switched on and off, because it can be considered geometrically that the curve shown in Figure 7b,c cannot be delineated if *t* is a constant value. *t* at b in Figure 7b is larger than *t* at a, *t* at d is larger than *t* at c, and *t* at e is larger than *t* at f. State e in Figure 7b corresponds to optical charging, and the state of f corresponds to optical discharging. 

**Figure 11:**
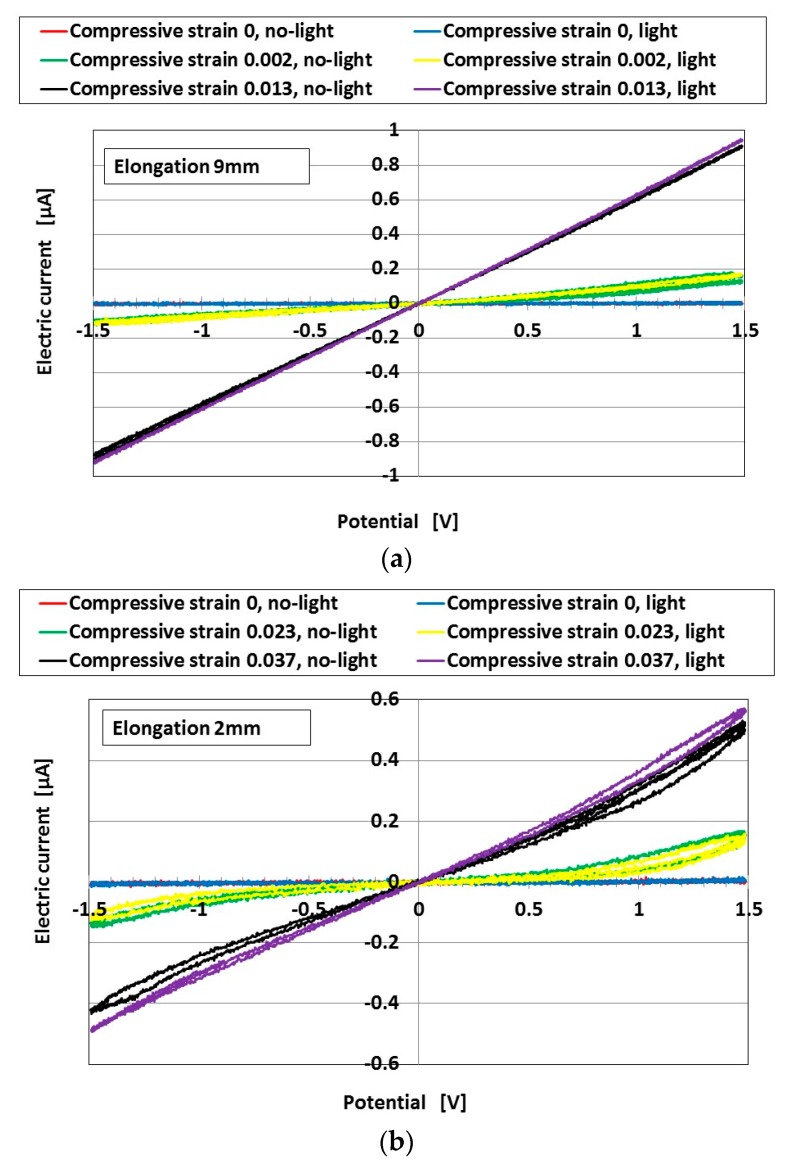
The effect of tension and compression on cyclic voltammetry plots measured by potentiostat at turning ultraviolet light on for wet-type MCF rubber solar cells electrolytically polymerized under a magnetic field with 0.22 g of Ruthenium complexes dye and 7.4 g of KI+I_2_ electrolyte: (**a**) elongation of 9 mm; (**b**) elongation of 2 mm. The MCF rubber had 3 g of TiO_2_, 6 g of Ni, 4.5 g of MF, and 9 g of NR-latex. The irradiation light was ultraviolet (40 Lux). The used experimental apparatus and procedure was the same as those used in the second report [19].

**Figure A5:**
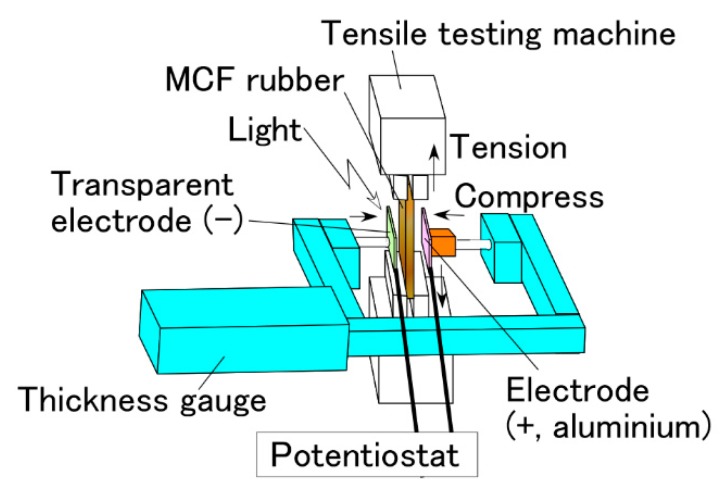
Schematic diagram of experimental apparatus for measuring cyclic voltammogram of wet-type MCF rubber solar cell under tension and compression.

The capacitance described above is in relation to charging and discharging by illumination. It is effective to measure the cyclic voltammogram of the MCF rubber solar cell in order to investigate the relation of capacitance to compression and elongation. The series of cyclic voltammogram plots in Figure 11 shows the relation between the electric current *I* and voltage *V* measured by potentiostat (HA-151B, Hokuto Denko Co. Ltd., Tokyo, Japan) and the *I*–*V* characteristics, at 50-mHz scan rates in the potential domain of −1.5–1.5 V, as shown by Figure A5 in Appendix B. The same experimental apparatus was used as the one for measuring photoelectricity under compression and elongation in the previous second report [19]. “No-light” in the figure denotes the case without irradiation, and “light” denotes the case with irradiation. The tensile strength to elongate the MCF rubber and compression with strain were simultaneously applied to the MCF rubber. At small elongation or compression, the *I*–*V* curve was nonlinear, and the area surrounded by the electric current and voltage was small. The *I*–*V* characteristics were different from those of an ordinary solid-state, dye-sensitized solar cell or polymer solar cell [12,13,22]. The MCF rubber solar cell is close to the *I*–*V* curve of a photodiode, as *I* becomes larger at the largest *V*. This is a typical property of MCF rubber solar cells, and it is suggested that it is possible to use the MCF rubber solar cell as a photodiode [23,24]. In particular, the curve becomes linear with increasing elongation or compression due to the heterojunction structure becoming deformed such that the distance between *A*^−^ and *D^+^* decreases. The linear tendency means that the MCF rubber solar cell becomes conductive.

On the other hand, from Figure 11, the area of hysteresis of the *I*–*V* curve becomes larger with increasing compressive strain, and then decreases with increasing compressive strain. The area decreases with increasing elongation. This tendency indicates that *C* becomes larger as the compression increases at small compressions, and then decreases with increasing elongation. The former tendency is coincident with the previously described results from Figure 2, Figure 7, Figure 8 and Figure 10. As shown in detail by Figure 11, the area of hysteresis is not different between charging and discharging. This tendency coincides with the result that *C R* is different between the states of charging and discharging, which can be derived from the previously described result in Figure 7b, wherein *t* at e is greater than *t* at f. 

**Figure 12:**
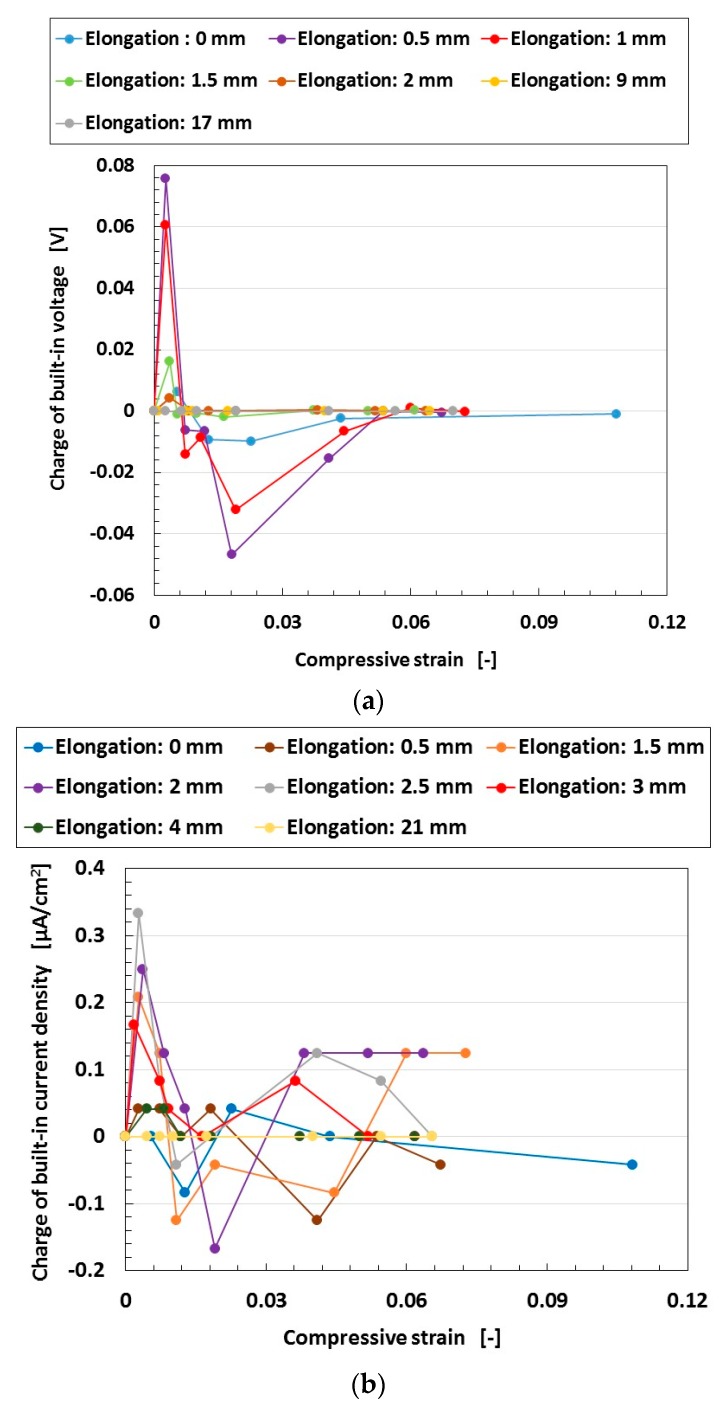
Charge of built-in electricity under compression and elongation at turning ultraviolet light on for a wet-type MCF rubber solar cell electrolytically polymerized under a magnetic field with 0.22 g of Ruthenium complexes dye and 7.4 g of KI+I_2_ electrolyte: (**a**) voltage; (**b**) current density. The MCF rubber had 3 g of TiO_2_, 6 g of Ni, 4.5 g of MF, and 9 g of NR-latex. The irradiation light was ultraviolet (40 lx). The experimental apparatus and procedure were the same as those used in the second report [19].

In conclusion, from the above-mentioned results, the wet-type MCF rubber solar cell under tension and compression can charge electricity. The voltage and current can be verified by other experimental results of charging, as shown by Figure 12. The electricity charged in the MCF rubber solar cell can be evaluated with the enhancement of built-in electricity (built-in voltage and built-in current) by irradiation. From the figure, the charge in the built-in electricity increases with increasing compression at a small compressive strain, and then decreases as the compressive strain increases further. This tendency coincides with the result of Figure 11. On the other hand, the charge in the built-in electricity increases at small elongations, and then decreases at larger elongations. The former tendency is found in the linear elastic region of the MCF rubber, and the latter at the plastic region. The former is a typical characteristic of the MCF rubber solar cell, and the MCF rubber solar cell might be used as an inner linear elastic region for engineering applications of H-Skin for robotics. The latter tendency coincides with the results of Figure 11.

## 3. MCF Rubber Battery

**Figure 13:**
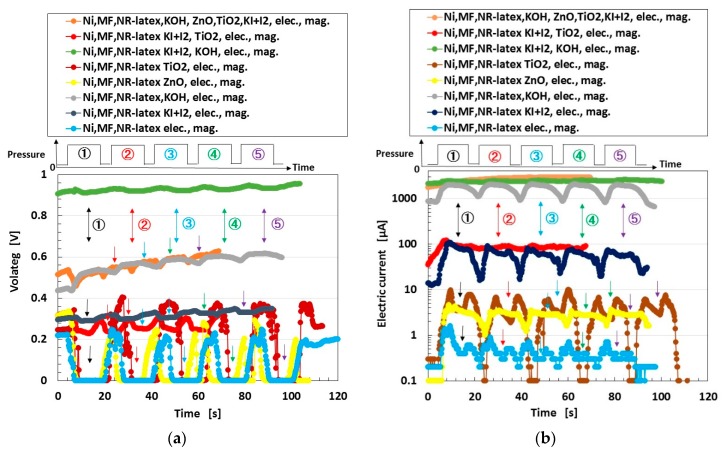
Change in voltage and electric current under five-times from ① to ⑤ repeated compression for MCF rubber electrolytically polymerized under a magnetic field: (**a**) voltage; (**b**) current density.

We investigated the electric charging by irradiation on an MCF solar cell rubber. As seen from Figure 11, the *I*–*V* characteristics at no-light can be suggested to have the same properties as those at irradiation, which is pointed out in Section 2. Therefore, there exists the possibility of an electric storage system in the MCF rubber under no-light. This is effective enough to be utilized for many engineering applications of H-Skin, because the MCF rubber can have an electric charge regardless of irradiation. In contrast to the expectation that the MCF rubber solar cell becomes mere MCF rubber in the experimental circumstance without irradiation, MCF rubber can have an electric charge in the case involving other agents except for sensitized dye, provided that it has an oxidation–reduction reaction such as that in the MCF rubber solar cell. Therefore, we used various kinds of agents and their compounds, as shown in Figure 13. The electric storage system of MCF rubber corresponds to built-in electricity. The present MCF rubber corresponds to dry-type MCF rubber.

The electrolytically polymerized MCF rubber was around 15 mm × 19 mm × 1 mm in size (“elec.” in the figure denotes electrolytic polymerization); a constant electric field was applied at 6 V, and an electric current of 2.7 A was passed between stainless-steel plates with a 1-mm gap for 10 min under atmospheric conditions, and a 188-mT magnetic field was applied across the liquid (“mag.” in the figure denotes application of the magnetic field). The rod with a diameter of φ8 mm attached on a small automatic measuring tensile testing machine (SL-6002, IMADA-SS, Co. Ltd., Toyohashi, Japan) that was used previously [19] was touched to one side of the aluminum electrodes between which the MCF rubber was sandwiched. The voltage and electric current between the electrodes were measured by a digital multi-meter (PC710, Sanwa Co. Ltd., Okayama, Japan). The figure shows the changes in voltage and electric current due to the application of step-like pressure with five repetitions from ① to ⑤. The arrows in the figure show the timing of the applied pressure. Each component of the used MCF rubber comprised 1.5 g of Ni, 0.75 g of MF, 4.5 g of NR-latex, 0.25 g of TiO_2_, 0.25 g of ZnO, 0.75 g of potassium hydroxide, KOH with 1 g water, and 0.5 g of KI+I_2_.

First, as seen from cases of “Ni, MF, NR-latex” and “Ni, MF, NR-latex, ZnO”, “Ni, MF, NR-latex, TiO_2_”, and “Ni, MF, NR-latex, KI+I_2_” (these cases are called “FC” for convenience), the voltage increased temporarily, and then decreased with increasing pressure, that is, it increased at lower pressure and decreased at higher pressure. The cause is due to the piezoelectric phenomenon that was clarified in the case of the “Ni, MF, NR-latex, ZnO” MCF rubber in the previous study [25]. At low pressure, the positive and negative ions approach each other such that the voltage increases. At higher pressure, the positive and negative ions come into contact, and the Ni particles aggregate such that the electron can pass through the MCF rubber; therefore, voltage decreases. On the other hand, in the other cases of MCF rubber in Figure 13 (these cases are called “LC” for convenience), even if the pressure was larger, the voltage changed according to the step-like shape of pressure. The cause was that the oxidation–reduction reaction in LC was larger than that in FC.

Next, regarding the electric current, in the case of FC, temporary decreasing can be seen. The cause is that the positive and negative ions come into contact and that electricity discharges. On the other hand, in the other case of LC, it changes according to the step-like shape of pressure.

Depending on the selection of the kinds of agents, we made an MCF rubber that had a larger amount of electricity that was comparable to an ordinary polymer battery: 1-V-order of voltage and 1-mA-order of electric current. The present MCF rubber is very flexible and elastic to compression and tension because of the NR-latex. The MCF rubber has such a small size and thickness, around 15 mm × 19 mm × 1 mm, that we can realize a lightweight battery. As a result, if we use many MCF rubbers, we can obtain a flexible and elastic battery with greater electric charging. Therefore, we can also realize H-Skin with electric charging that does not require irradiation.

## 4. Conclusions

We clarified the feasibility of the autonomous usage of elastic and compressible H-Skin without any battery or external power generator for electric charging for situations with and without irradiation by indwelling in the MCF rubber. By using tunneling theory and the equivalent electric circuit on-resistance and capacitance, and by comparing with the experimental data of resistance, voltage, and electric current at irradiation to those of the cyclic voltammogram, it was clarified that the wet-type MCF rubber solar cell under tension and compression can charge electricity. In the case without irradiation, the MCF rubber can also store electricity, which corresponds to the built-in electricity. In conclusion, as for the electric charge of MCF rubber, regarding Section 2, the optimal condition was electrolytically polymerized MCF rubber with sensitized dye and electrolyte for photovoltaics. On the other hand, regarding Section 3, the optimal condition was KOH for battery.

Depending on the selection of agent type, we can make an MCF rubber with greater built-in electricity and lighter weight than previously described. For example, it will be effective for renewable-energy batteries, including those for wind and solar power, because the current battery has serious problems due to its heavy weight and large size. In addition, the present MCF rubber is elastic and compressible. Therefore, we can propose many engineering applications that utilize MCF rubber in many fields. The important application of MCF rubber is electric charge restored inner an elastic, compressible, and deformable rubber, except for sensing. For example, if the artificial skin as the husk of a robot is installed on the robot, it is similar to human skin enough to be familiar to us, and may become a robotics style in the future, since the artificial skin is elastic rubber enough to be deformable for bending and compression. In addition, electricity can be charged in itself by irradiation, and so the robot does not need any electric power supply or battery. Even if not by illumination, it does not also need any electric power supply because of the built-in electricity as battery. Furthermore, as the rubber has also sensing as shown by the consecutive first and second reports, the robot can be sensible to force and temperature such as human skin. The robot might work in space as an astronaut, and it can be charged in itself by irradiation from the sun and manipulate any instruments by sensing. Many ideas can be realized with many other fields as well as within robotics.

## Figures and Tables

**Figure A1:**
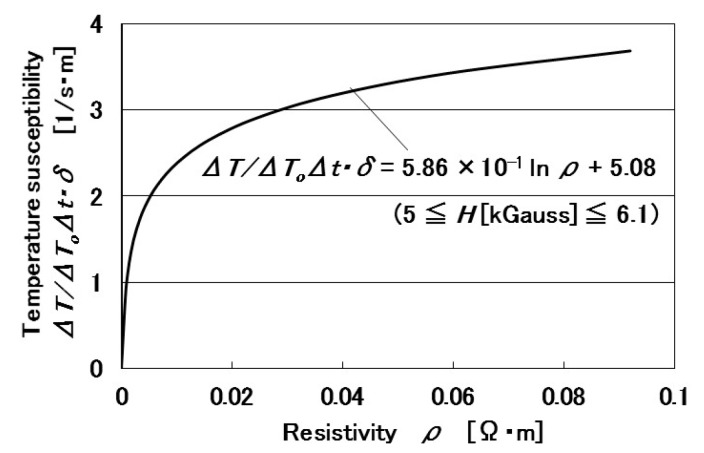
Relation between temperature susceptibility and resistivity. Δ*T* is the change in temperature of dry-type MCF rubber by touching to a heated body, Δ*t* the response time of temperature at heating, Δ*T_o_* the enhancement of temperature applied by heating, *δ* the thickness of MCF rubber.

**Figure A2:**
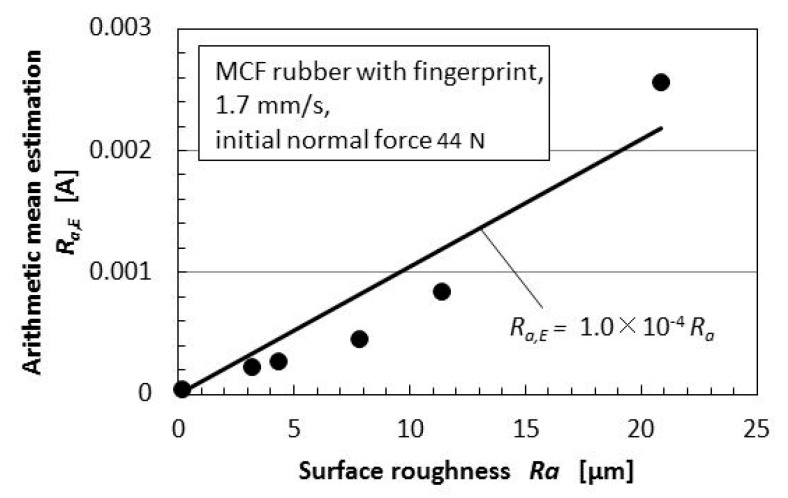
Relation between the parameter estimated of surface roughness of a body and the one of electric current curve measured by dry-type MCF rubber under rubbing speed and pressing force which is presented as “initial normal force” in the figure [27].

**Figure A3:**
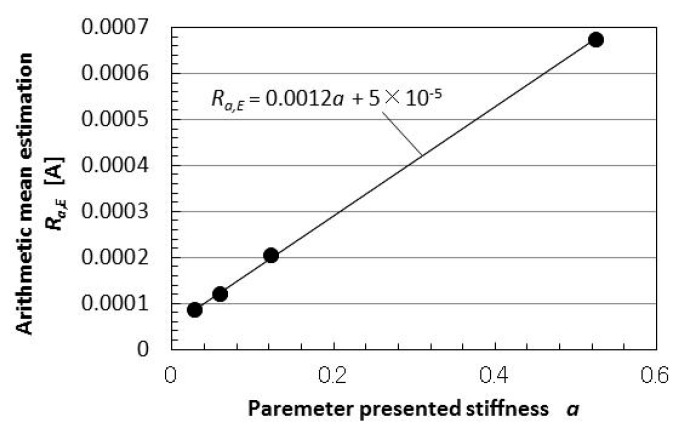
Relation between the parameter estimated of surface roughness of soft body and the softness parameter *a* [28].

**Figure A4:**
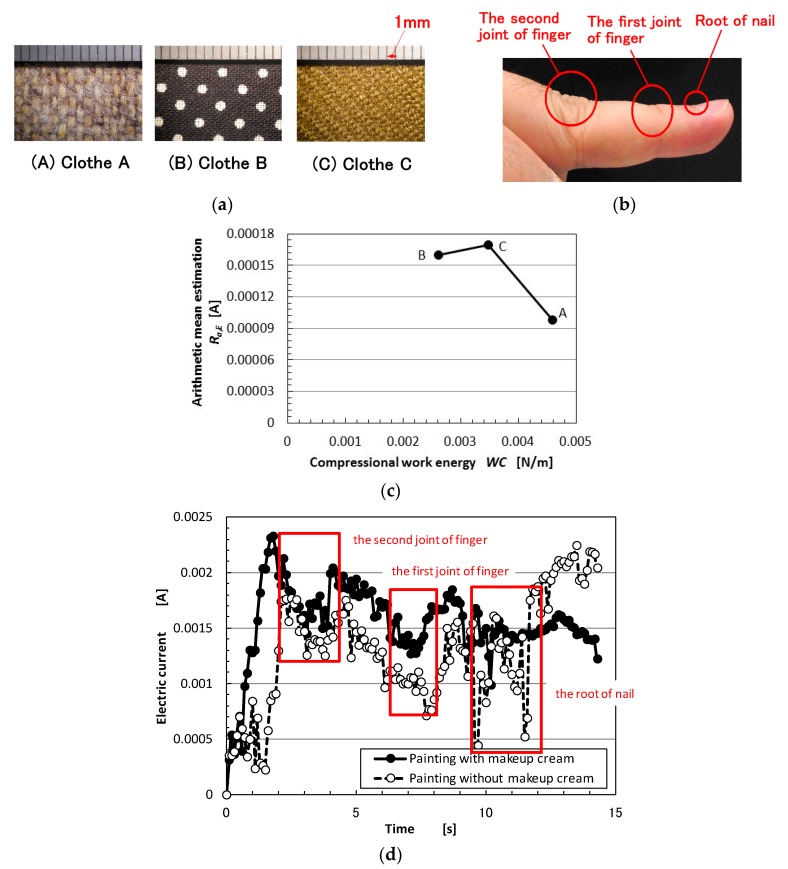
Photographs and results of measured surface roughness of varied body with softness by dry-type MCF rubber: (**a**) the photographs of cloth; (**b**) the photograph of finger; (**c**) the results in the case of (**a**); (**d**) the results in the case of (**b**) [28].

## Nomenclature

*a*	softness parameter [N/m]
*C*	capacitance [F]
*C**	dimensionless capacitance [-]
*C_n_**	dimensionless capacitance located at *n*’s turn cell [-]
*cs*	compressive strain [-]
*e*	elementary charge [C]
*eE_o_*	applied voltage [eV]
*f*	frequency of light [1/s]
*H*	magnetic field strength [gauss]
*h*	Planck’s constant [Js]
*I*	current density [A]
*I_d_*	diode current [A]
*I_ph_*	photocurrent [A]
*I_r_*	light intensity [cd]
*I_S_*	reverse-bias saturation current [A]
*I_sh_*	shunt current [A]
*k*	Boltzmann constant [J/K]
*k_1_*	glass factor [1/m]
*m*	mass of electron [kg]
*N*	recombination in the *n*–*p*-type semiconductor [1/m^3^]
*N_d_*	diode ideality factor [-]
*n*	pairs of regions [-]
*R*	resistance [Ω]
*R**	dimensionless resistance [-]
*R_a_*	arithmetical mean deviation of the assessed profile of surface roughness [m]
*R_a,E_*	arithmetical mean deviation of the assessed profile of electric current by sensing [A]
*R_s_*	series resistance [Ω]
*R_sh_*	shunt resistance [Ω]
*R_y_*	maximum height of the assessed profile of surface roughness [m]
*R_y,E_*	maximum height of the assessed profile of electric current by sensing [A]
*R_z_*	Japanese industrial standard based on the five highest peaks and lowest valleys over the entire sampling length of the assessed profile of surface roughness [m]
*R_z,E_*	Japanese industrial standard based on the five highest peaks and lowest valleys over the entire sampling length of the assessed profile of electric current by sensing [A]
*R_∞_*	representative resistance [Ω]
*T*	transmitted probability [-]
*T_a_*	absolute temperature [K]
*T_i_*	transmitted probability at *i*-ordered pair of regions [-]
*T_out_*	transmitted probability at the outlet of *n* pairs of regions [-]
*t*	response time [s]
*t**	dimensionless time [-]
*t_1_*	response time based on *C* and *R* [s]
*t_2_*	response time based on the speed of carrier diffusion at deeper regions than the depletion layer [s]
*t_3_*	response time based on the speed of carriers in the inner depletion layer [s]
*V*	voltage [V]
*V_D_*	diffusion potential [V]
*V_f_*	forward bias voltage [V]
*V_i_*	voltage drop at the thin isolated layer [V]
*V_o_*	potential energy [J]
*WC*	compressional work energy [N/m]
*α*	optical absorption coefficient [1/m]
Δ*T*	change in temperature of dry-type MCF rubber by touching to a heated body [K]
Δ*t*	response time of temperature at heating [s]
Δ*T_o_*	enhancement of temperature applied by heating [K]
*δ*	thickness of MCF rubber [m]
*ε*	energy of the electron [J]
*ε’*	dielectric constant of the MCF rubber solar [-]
*γ*	wave number [1/m]
*γ_i_*	wave number at *i*-ordered pair of regions [1/m]
*ħ*	Dirac’s constant (=*h*/2π) [Js]

## Appendix A

In the basic study for the purpose of the engineering application of H-Skin, we have investigated temperature susceptibility [26]. The MCF rubber was produced by a dry method in which the rubber is solidified in ambient under a constant temperature. Therefore, this was named dry-type MCF rubber. The temperature susceptibility can be evaluated by the changes in the resistance of the MCF rubber, as shown by Figure A1, in the case where the MCF rubber touches a heated surface made of silicon oil rubber of dimensions 15 mm × 20 mm × 0.5 mm consisting of 3 g Cu, 3 g Ni, and 4 g MF with 50 wt % Fe_3_O_4_ (HC-50, Ichinen-Chemicals Co., Ltd., Shibaura, Japan), and 10 g silicon oil rubber (SH9550, Shin-Etsu Chemical Co. Ltd., Tokyo, Japan) solidified in the air under the application of a magnetic field *H*.

On the other hand, we have challenged in sensing of many varied body’s surfaces related to shearing motion. At first, we can evaluate the surface roughness by rubbing a finger sack on which MCF rubber is attached [27]. This method of measuring surface roughness is novel without using ordinary surface roughness meter and effective enough to easily measure the surface roughness of large aircraft, ship, automobile, etc. By introducing parameters *R_a_*, *R_y_*, *R_z_*, etc. used ordinarily in the case of estimating surface roughness at the field of precision, the electric current curve measured by MCF rubber is calculated by the equations of these parameters, *R_a,E_*, *R_y,E_*, *R_z,E_*, etc. *R_a,E_*, *R_y,E_*, *R_z,E_*, etc. have algebraic relation to the surface roughness of the body, *R_a_*, *R_y_*, *R_z_*, etc., as shown by Figure A2, and a function of rubbing speed and pressing force on the body so that anyone can similarly obtain the surface roughness although rubbing speed and pressing force depend on the person’s action. The dry-type MCF rubber was made of NR-latex with around 19 mm × 24 mm × 0.7 mm consisting of 20 g Cu, 40 g Ni, 15 g MF with 35 wt % Fe_3_O_4_ (W-35, Ichinen-Chemicals Co., Ltd., Shibaura, Japan), 80 g NR-latex (Rejitex Co., Ltd., Atsugi, Japan) solidified in the air under the application of a magnetic field strength of 560 mT.

Second, the dry-type MCF rubber can estimate softness, such as silicon oil gel. As shown by Figure A3, the parameters *R_a,E_*, in addition, *R_y,E_*, *R_z,E_*, etc., have a function of the softness evaluated by a related to the stiffness, softness parameter *a*, which depends on compressibility and obtained from the relation between the pressing force and compressive displacement [28]. The MCF rubber used was the same as that in Figure A2.

Third, the dry-type MCF rubber can measure the surface roughness of a varied body with paper or cloth in Figure A4a, or skin on a finger in Figure A4b. The parameters for estimating the softness and roughness of cloth have been generally proposed WC which is usually used in the field of sensing of its texture, as shown in Figure A4c. The difference in softness and roughness by different kinds of cloth cannot be evaluated by compressional work energy WC which is ordinarily used in the field of measuring softness of a body and means energy required for compression, but by the parameters, *R_a,E_*, *R_y,E_*, *R_z,E_*, etc. on MCF rubber. Regarding Figure A4b, the smoothness of skin by cosmetics can be estimated, and the electric currents of MCF rubber by rubbing on the skin painted both with and without makeup cream are shown in Figure A4d [28]. The MCF rubber used was the same as that in Figure A2.

## Appendix B

We used the same experimental apparatus of compression and elongation as the one in the previous 2nd report [19]. The MCF rubber electrolytically polymerized with 0.22-g Ruthenium complexes dye on one side and 7.4-g KI+I_2_ on the other was set on a small automatic measuring tensile testing machine (SL-6002, IMADA-SS Co. Ltd., Toyohasi, Japan) as shown in Figure A5. A transparent glass (20 mm × 30 mm) coated with TiO_2_ was irradiated with ultraviolet light and connected to the cathode of the solar cell. The MCF rubber was elongated vertically by the tensile testing machine, and at the same time compressed by a thickness gauge transverse to the rubber. For compression, the irradiated glass was compressed by a cylinder with φ6.5 mm connected to the thickness gauge. Therefore, the irradiated area of the MCF rubber was the area that remained outside of its circular area. The irradiation light was ultraviolet (40 Lux).

## References

[1] Yoshida T. (2005). The Leading Edge of Development of Super Five Senses Sensor.

[2] Dargahi J. (2002). An endoscopic and robotic tooth-like compliance and roughness tactile sensor. Trans. ASME J. Mech. Des..

[3] Tanaka M., Tanaka Y., Chonan S. (2008). Measurement and evaluation of tactile sensations using a PVDF sensor. J. Intell. Mater. Syst. Struct..

[4] Canepa G., Rossi D.D., Mageces G., Germagnoli F., Caiti A., Parisini T. (1993). Skin-like tactile sensor arrays for contact stress field extraction. Mater. Sci. Eng. C.

[5] Sato K., Kawakami N., Kamiyama K., Tachi S. (2010). Finger-shaped gelforce: Sensor for measuring surface traction fields for robotic hand. IEEE Trans. Haptics.

[6] Shimada K., Zheng Y., Saga N. (2014). Experimental investigation on technique to read convex shape by MCF rubber sensor utilizing robot action. J. Jpn. Soc. Exp. Mech..

[7] Nomata T., Okuyama T., Tanaka M. (2011). Quantification of a constant stimulus applied to infants by diapers–Relationship between walking motion and constant stimuli. J. Soc. Appl. Electromagn. Mech..

[8] TianKhoon L., Hassan N.H., Rahman M.Y.A., Vedarajan R., Matumi N., Ahmad A. (2015). One-pot synthesis nano-hybrid ZrO2-TiO2 fillers in 49% poly(methyl methacrylate) grafted natural rubber (MG49) based nano-composite polymer electrolyte for lithium ion battery application. Solid Sate Ion..

[9] Ali A.M.M., Subban R.H.Y., Bahron H., Yahya M.Z.A., Kamisa A.S. (2013). Investigation on modified natural rubber gel polymer electrolytes for lithium polymer battery. J. Power Sources.

[10] Won S.C., Sung H.A., Harim J., Yong G.S., Jong H.K. (2012). Rubbery copolymer electrolytes containing polymerized ionic liquid for dye-sensitized solar cells. J. Solid State Electrochem..

[11] Dong J.K., Sang J.K., Dong K.R., Jong H.K. (2013). Synthesis of low-cost, rubbery amphiphilic comb-like copolymers and their use in the templated synthesis of mesoporous TiO_2_ films for solid-state dye-sensitized solar cells. Phys. Chem. Chem. Phys..

[12] Suleman M., Kumar Y., Hashmi S.A. (2015). Solid-state electric double layer capacitors fabricated with plastic crystal based flexible gel polymer electrolytes: Effective role of electrolyte anions. Mater. Chem. Phys..

[13] Li Z., Ma G., Ge R., Qin F., Dong X., Meng W., Liu T., Tong J., Jiang F., Zhou Y. (2016). Free-standing conducting polymer films for high-performance energy devices. Angew. Chem. Int. Ed..

[14] Song H., Cai K. (2017). Preparation and properties of PEDOT:PSS/Te nanorod composite films for flexible thermoelectric power generator. Energy.

[15] Chen C.P., Chiang C.Y., Yu Y.Y., Hsiao Y.S., Chen W.C. (2017). High-performance, robust, stretchable organic photovoltaics using commercially available tape as a deformable substrate. Solar Energy Mater. Solar Cells.

[16] Park J.I., Heo J.H., Park S.H., Hong K.I., Jeong H.G., Im S.H., Kim H.K. (2017). Highly flexible InSnO electrodes on thin colourless polyimide substrate for high-performance flexible CH_3_NH_3_PbI_3_ perovskite solar cells. J. Power Sources.

[17] Shimada K. (2018). Elastic MCF rubber with photovoltaics and sensing for use as artificial or hybrid skin (H-Skin): 1st report on dry-type solar cell rubber with piezoelectricity for compressive sensing. Sensors.

[18] Hammock M.L., Chortos A., Tee B.C.K., Tok J.B.H., Bao Z. (2013). 25th anniversary article: The evolution of electronic skin (E-Skin): A brief history, design considerations, and recent progress. Adv. Mater..

[19] Shimada K. (2018). Elastic MCF rubber with photovoltaics and sensing on hybrid skin (H-Skin) for artificial skin by utilizing natural rubber: 2nd report on effect of tension and compression on properties of hybrid photo- and piezo-electricity in wet-type solar cell rubber. Sensors.

[20] Shimada K. (2017). Enhancement of MCF rubber utilizing electric and magnetic fields, and clarification of electrolytic polymerization. Sensors.

[21] Sasabe H. (1988). Mechanisms of photo-electronic energy conversion in organic materials and their application. Chem. Chem. Ind..

[22] Rastogi A.C., Janardhana N.R. (2014). Properties of CuSbS_2_ thin films electrodeposited from ionic liquids as p-type absorber for photovoltaic solar cells. Thin Solid Films.

[23] Buffa M., Carturan S., Debije M.G., Quaranta A., Maggioni G. (2012). Dye-doped polysiloxane rubbers for luminescent solar concentrator systems. Solar Eng. Mater. Solar Cells.

[24] Nyberg T., Zhang F., Inganas O. (2002). Macromolecular nanoelectronics. Curr. Appl. Phys..

[25] Shimada K., Saga N. (2017). Development of a hybrid piezo natural rubber piezoelectricity and piezoresistivity sensor with magnetic clusters made by electric and magnetic field assistance and filling with magnetic compound fluid. Sensors.

[26] Shimada K., Zheng Y. (2007). Development of MCF rubber with temperature and electric senses for an element material in haptic robot sensor. Trans. Jpn. Soc. Mech. Eng..

[27] Shimada K., Hayasaka T. (2012). Principle and characteristics of high sensitive haptic MCF rubber perceptible to shear force as well as normal force utilizing intelligent fluid. J. Jpn. Soc. Exp. Mech..

[28] Shimada K., Hayasaka T. (2012). Investigation of applicability of high sensitive haptic MCF rubber perceptible to shear force as well as normal force utilizing intelligent fluid. J. Jpn. Soc. Exp. Mech..

